# Evaluation of the feasibility of artificial intelligence models as patient information advisors for human papillomavirus vaccines

**DOI:** 10.1590/1806-9282.20250809

**Published:** 2025-12-08

**Authors:** Abdulkadir Kaya, Hüseyin Nejat Kücükdag, Betül Keyif

**Affiliations:** 1 Düzce University, Faculty of Medicine, Department of Family Medicine – Düzce, Turkey.; 2 Düzce University, Faculty of Medicine, Department of Obstetrics and Gynecology – Düzce, Turkey.

**Keywords:** Artificial intelligence, HPV vaccine, Patient education, Chatbots, Health communication

## Abstract

**OBJECTIVE:**

Artificial intelligence chatbots are increasingly used to disseminate health information. The aim of this study was to evaluate the accuracy, reliability, quality, and readability of responses generated by four artificial intelligence chatbots regarding human papillomavirus vaccination.

**METHODS:**

Frequently asked questions about human papillomavirus vaccination were identified using a Google search tool, and these questions were posed to the ChatGPT-3.5, Gemini, Copilot artificial intelligence, and ChatGPT-4 models. Responses were assessed for accuracy (five-point Likert scale), reliability (modified DISCERN scale), quality (Global Quality Scale), and readability (Flesch Reading Ease Score). Interobserver agreement was evaluated by the intraclass correlation coefficient. The results were evaluated at a significance level of p<0.05. SPSS 23.0 package programme was used in the analyses.

**RESULTS:**

There were significant differences between chatbots in terms of accuracy (p=0.001), reliability (p<0.001), and quality (p<0.001), but no significant difference in readability (p=0.497). ChatGPT-4 demonstrated the highest accuracy and quality, while Copilot artificial intelligence demonstrated superior reliability. All models produced responses that were moderately difficult to read. The intraclass correlation coefficient values for inter-rater reliability ranged from 0.034 to 0.512.

**CONCLUSION:**

Artificial intelligence chatbots show promising potential for use as patient information counsellors regarding human papillomavirus vaccination. However, improvements in readability and consistent evidence-based content generation are required before widespread clinical application.

## INTRODUCTION

Artificial intelligence (AI), first defined in 1956, refers to systems that can perform cognitive functions that require human intelligence, such as decision making, problem solving, and learning^1^. Machine learning and large language models, which are among the subfields of AI, are used in healthcare services, especially medicine and dentistry^1,2^. These models, trained with deep learning methods, have human-like text production and interpretation skills^1^.

There are many language models with different features today. ChatGPT, based on GPT-3.5, is available free of charge, while the more advanced GPT-4 is only available with a paid subscription^3^. Microsoft's Copilot (formerly Bing Chat) and Google's Gemini (formerly Bard) models are also available in a similar way. In particular, Copilot's additional capabilities such as internet access, up-to-date information presentation, and citation are noteworthy^3,4^.

AI-powered chatbots stand out with their advantages of accessibility and rapid provision of information for patients; however, their potential to produce false, irrelevant, or misleading content poses serious risks in critical areas such as healthcare^5^. Therefore, the reliability, content quality, and accuracy of the information provided must be checked with physician approval.

Human papillomavirus (HPV) is a common infection that can cause various types of cancer, especially cervical cancer. HPV vaccines are an effective and safe preventive health practice in preventing this infection and related diseases^6,7^. Issues such as the duration of immunity, safety, age group to which the vaccine will be applied, and its integration into national vaccination strategies are important for public health. The use of HPV vaccines together with screening programs is important for the integration of primary and secondary prevention against cancer. In this context, raising awareness among society and health professionals and developing and disseminating vaccine policies based on scientific data are of critical importance for protecting public health^8^. The World Health Organization recommends that vaccination be administered, especially to the 9–14 age group^9^. The quadrivalent HPV vaccine is available in immunization programmes in 64 countries worldwide, including Brazil^10^. However, lack of information, vaccine hesitation, and access problems may negatively affect vaccination rates^10,11^.

The internet is the first source that most patients turn to when seeking health-related information^12^. This makes the accuracy and reliability of chatbots trained on large text data sets even more important^13,14^. However, the limitations of medical expertise and the risk of hallucination production and misinformation of these systems should be taken into account, and it should not be forgotten that answers should be verified by experts, especially in health-related areas^5,14^.

It was hypothesized that different AI models would show significant differences in terms of accuracy, reliability, content quality, and readability when providing patient information about the HPV vaccine. In this context, this study aims to compare the patient information capabilities of common AI-based chatbots such as ChatGPT-3.5, ChatGPT-4, Gemini, and Copilot regarding HPV vaccine and is the first in the literature. The findings will reveal the potential contributions and limitations of AI in information processes for healthcare professionals and will also guide public health practices and health literacy.

## METHODS

This study was approved by Düzce University Faculty of Medicine Ethics Committee (Decision no: 2025/89, Approval Date: 24.03.2025). It was hypothesized that different AI models would show significant differences in terms of accuracy, reliability, content quality, and readability when providing patient information about the HPV vaccine. To test this hypothesis, responses generated by four AI models were evaluated by two independent experts based on these four criteria. The study measured the accuracy, reliability, quality, and readability levels of artificial intelligence models such as ChatGPT-3.5, Gemini, Copilot AI, and ChatGPT-4. The 45 most frequently asked questions about HPV vaccines were determined via Google. It is known that AI programs can work in different capacities in different languages. Therefore, the questions were asked and evaluated in Turkish.

A search was conducted using the Google search tool to identify websites that responded to the search term "frequently asked questions about HPV vaccines." According to a survey, 57% of patients prefer to turn to the internet first for health-related information^15^. The literature reports that 90% of search engine users only view the first three pages of search results^12^. Therefore, the first 30 sites were analyzed, and a pool of 168 questions was created by excluding "irrelevant," "duplicate," non-Turkish, and "sponsored advertising" sites. The 45 questions most frequently asked by the authors were included, while questions that were repetitive, similar, irrelevant, and included sponsored advertising were excluded.

The tested chatbots were ChatGPT model GPT-3.5, which is currently used free of charge, Gemini and Copilot chatbots, and ChatGPT model GPT-4, which can be used with a paid subscription. Questions were directed to these four different artificial intelligence models, and answers were received. These models were logged in with different accounts so that they would be used for the first time. The answers were evaluated and scored by two family medicine experts according to the criteria of Accuracy (Five-Point Likert Scale), Reliability (Modified DISCERN Scale), Quality (Global Quality Scale), and Readability (Flesch Reading Ease Score).

A modified version of the DISCERN instrument was used to evaluate the quality and reliability of the AI-generated responses. The original DISCERN tool is a validated questionnaire developed to assess the quality of written consumer health information, particularly about treatment choices. In this study, a simplified version was adapted, focusing on core domains such as clarity, source citation, balance/unbiased information, and overall reliability. The modified version consisted of five items rated on a 5-point Likert scale (1=low quality, 5=high quality)^16^.

### Statistical analysis

Numeric data were described using the mean, standard deviation (SD), median, and interquartile range (IQR). For comparisons between the groups, Kolmogorov-Smirnov test was used to verify the normality of distribution; The Kruskal-Wallis test was used to observe non-normal distribution of the data and the violation of the homogeneity of variances assumption; the Tamhane's test, which does not assume equal variances, was applied for post-hoc analysis; interobserver agreement was evaluated by intraclass correlation coefficient (ICC). The results were evaluated at a significance level of p<0.05. SPSS 23.0 package programme was used in the analyses.

## RESULTS

A total of 45 questions were asked in the study. The accuracy, reliability, quality, and readability scores of the chatbots’ responses to the questions are shown in Table 1. It was observed that Accuracy (p=0.001), Reliability (p<0.001), and Quality (p<0.001) scores were significantly different in all chatbots. Readability scores were not significantly different among chatbots (p=0.497) (Table 1).

**Table 1:** A comparison of the Likert accuracy, reliability, quality, and readability scores of four different chatbots.

		Chatbots				p*
		C1	C2	C3	C4	
Accuracy	Mean±SD	4.66±0.52	4.33±0.70	4.49±0.62	4.68±0.52	**0.001**
	Median (IQR)	5 (1)	4 (1)	5 (1)	5 (1)	
Reliability	Mean±SD	4.31±0.51	4.01±0.66	4.42±0.62	4.33±0.56	**<0.001**
	Median (IQR)	4 (1)	4 (0)	4 (1)	4 (1)	
Quality	Mean±SD	4.28±0.60	3.96±0.67	3.99±0.59	4.63±0.53	**<0.001**
	Median (IQR)	4 (1)	4 (0)	4 (0)	5 (1)	
Readability	Mean±SD	82.94±8.96	82.50±9.28	80.72±9.90	81.06±9.07	0.497
	Median (IQR)	80 (10)	85 (10)	80 (20)	80 (20)	

C1: ChatGPT-3.5; C2: Gemini; C3: Copilot AI; C4: ChatGPT-4; SD: standard deviation; IQR: interquartile range.

* Kruskal-Wallis analysis. The significant values are presented in bold.

In the post-hoc analysis, the significances between the groups are given in Table 2. In terms of Accuracy, Gemini was significantly lower than ChatGPT-3.5 and ChatGPT-4; no significant difference was observed among the others. The highest Accuracy value was in ChatGPT-4. In terms of Reliability, Gemini and ChatGPT-3.5 were significantly lower than Copilot AI and ChatGPT-4; the others were not significantly different. The highest Reliability value was in Copilot AI. In terms of Quality, Gemini and Copilot AI were not significantly different; others were significantly different. The highest Quality value was in ChatGPT-4 (Tables 1 and 2).

**Table 2:** Post-hoc pairwise comparison of scores in artificial intelligence chatbots.

Chatbots	Chatbots	Accuracy	Reliability	Quality
C1	C2	**0.004**	**0.005**	**0.005**
	C3	0.281	0.719	**0.008**
	C4	1.000	1.000	**<0.001**
C2	C1	**0.004**	**0.005**	**0.005**
	C3	0.529	**<0.001**	1.000
	C4	**0.001**	**0.003**	**<0.001**
C3	C4	0.156	0.895	**<0.001**

C1: ChatGPT-3.5; C2: Gemini; C3: Copilot AI; C4: ChatGPT-4. Tamhane post-hoc test was used. The significant values are presented in bold.

The ICC values for inter-rater reliability ranged from 0.034 to 0.512. For Accuracy, both observers gave significantly correlated responses. For Reliability, both observers gave significantly correlated responses for Gemini and Copilot AI. For Quality, both observers gave significantly correlated responses for ChatGPT-3.5, Gemini, and Copilot AI. For Readability, both observers gave significantly correlated responses for ChatGPT-3.5, Gemini, and ChatGPT-4 (Table 3).

**Table 3:** The intraclass correlation coefficient of evaluators’ data.

Criteria	Chatbots	Intraclass correlation coefficient 95%CI	p
Accuracy	C1	0.391 (0.113–0.612)	**0.004**
	C2	0.424 (0.159–0.634)	**0.001**
	C3	0.500 (0.244–0.691)	**<0.001**
	C4	0.374 (0.093–0.600)	**0.005**
Reliability	C1	0.110 (-0.110 to 0.346)	0.165
	C2	0.471 (0.211–0.669)	**<0.001**
	C3	0.423 (0.156–0.634)	**0.002**
	C4	0.131 (-0.082 to 0.364)	0.051
Quality	C1	0.246 (-0.034 to 0.494)	**0.043**
	C2	0.512 (0.265–0.697)	**<0.001**
	C3	0.347 (0.075–0.574)	**0.007**
	C4	0.163 (-0.137 to 0.435)	0.142
Readability	C1	0.235 (-0.029 to 0.479)	**0.034**
	C2	0.301 (-0.030 to 0.565)	**0.002**
	C3	0.034 (-0.071 to 0.180)	0.276
	C4	0.275 (-0.098 to 0.612)	**<0.001**

C1: ChatGPT-3.5; C2: Gemini; C3: Copilot AI; C4: ChatGPT-4; CI: confidence interval. The significant values are presented in bold.

## DISCUSSION

In today's world, where the use of AI agents is rapidly increasing, patients and healthy people are questioning health issues on the internet, especially with AI. The accuracy, reliability, quality, and readability of the responses generated by four different AI chatbots regarding HPV vaccination information were evaluated. The findings revealed that there were statistically significant differences between AI models in terms of accuracy, reliability, and quality scores, while readability scores were similar between the models. It is also a fact that the AI models used mostly provided sufficient answers to questions asked about HPV vaccines, but they had deficiencies according to the sources that expert evaluators relied on in terms of accuracy, reliability, quality, and readability.

Accuracy received the highest scores in the responses given by chatbots to questions, both in all robots and according to other criteria. In similar studies, robots generally received higher scores in terms of accuracy^2,17^. From this, it can be thought that although the medical information provided by chatbots is accurate, they may fall behind in terms of Reliability, Quality, and Readability.

ChatGPT-4 achieved the highest accuracy and quality scores, while Gemini performed significantly lower in these areas. In terms of reliability, Copilot AI outperformed the others, while Gemini again showed slightly lower results. One possible reason for Copilot's higher reliability scores is its ability to generate responses that include verifiable references from recent and reputable sources. Unlike other AI models evaluated in this study, Copilot occasionally cited up-to-date sources and provided URLs or reference identifiers, which may have contributed to the experts’ perception of greater trustworthiness. In contrast, models such as ChatGPT tended to offer more general statements without source attribution. This suggests that the ability to link responses to identifiable sources may be a critical factor in enhancing perceived reliability in AI-based patient information tools. These results are consistent with previous studies suggesting that new large language models such as GPT-4 tend to provide more accurate and higher quality health information on vaccination than previous models^18^. Similar evaluations in the field of orthodontics found that GPT-4 and Copilot outperformed Gemini and GPT-3.5, especially in terms of accuracy and quality of health-related information^17^. Consistent with their findings, our results also show that the updated models generally provide better healthcare answers. ChatGPT-4 is significantly superior in terms of Accuracy, Reliability, and Quality when evaluating answers to frequently asked questions about HPV vaccines.

However, despite significant progress, some limitations remain. Our analysis showed that while accuracy, reliability, and quality varied among chatbots, readability levels across models remained similar, and Flesch Readability Scores generally indicate that the text is fairly easy to understand, but that people with the lowest level of education may not be able to comprehend it. This finding is important because patient reading comprehension plays a critical role in making informed health decisions^19^. The literature has emphasized that even when information is accurate, high linguistic complexity can hinder effective transfer of information, especially in populations with low health literacy^20^. In their study, Costa et al. showed that even Brazilian medical students had low levels of knowledge about the HPV vaccine and recommended promoting information, counseling, and continuous education as a strategy to increase acceptance of the vaccine, ensure its implementation, and guarantee its effectiveness in reducing future cases of cervical cancer^21^. Gomes et al. also directly identified factors influencing knowledge and acceptance of the HPV vaccine among Brazilian adolescents and parents in their study, highlighting barriers such as misinformation, fear, and lack of doctor recommendation^10^. In this regard, the linguistic comprehensibility and accessibility of AI tools as information sources are also important.

Another important observation from this study was that while intraclass correlation was adequate for accuracy, intraclass correlation was low for some chatbots for other criteria. This suggests that this may be influenced by the professional experience of the raters as well as their sources of information. This has been emphasized in other studies^22,23^.

Strengths of our study include comprehensive assessment across multiple performance dimensions (accuracy, reliability, quality, readability) and the inclusion of four different AI models. In addition, the use of validated scales such as the modified DISCERN, Global Quality Scale (GQS), and Flesch Reading Ease Score adds methodological robustness to the study. To the best of our knowledge, this is the first study to compare the accuracy, reliability, quality, and readability of responses from four different AI models regarding HPV vaccine information for patients. While previous studies have focused on single models or general medical queries, this study provides a direct comparison in a specific public health domain.

Previous studies have assessed the performance of individual AI models such as ChatGPT in delivering medical information, often reporting high readability but variable accuracy^24,25^. Our findings align with these studies in terms of readability but also highlight significant inter-model variability, particularly in the reliability and source referencing of responses.

An important limitation of this study is that all interactions with AI models were conducted in Turkish. Language plays a critical role in the performance of large language models, especially in the field of healthcare communication. As a result, the findings may not fully reflect how these models perform in Portuguese or other languages. Therefore, the applicability of these results to other world languages is limited. Second, the assessment included a certain number of HPV vaccine–related questions; the subject may not have been fully interrogated with these questions. Third, although two independent experts evaluated the responses, subjective interpretation cannot be completely eliminated.

Future research should investigate the use of AI-generated educational content in real-world patient populations and examine its real effects on patient knowledge, attitudes, and vaccination uptake. Additionally, improvements in the readability of AI outputs remain critical for broader accessibility and effectiveness, particularly among individuals with limited health literacy.

## CONCLUSION

This study demonstrated that AI chatbots have significant potential to provide accurate, reliable, and quality information on HPV vaccination. Among the evaluated models, ChatGPT-4 achieved the highest accuracy and quality scores, while Copilot AI demonstrated the best reliability. However, readability levels remained moderate across all chatbots, and improvements are clearly needed in this area. Despite these promising findings, the application of AI chatbots as autonomous patient education tools remains limited due to their variable performance across metrics and the complexity of the language used. While AI is used for patient health information, it is of great importance for society to reach primary health care services as the first point of access for vaccination. Future efforts should focus on improving the readability and evidence-based quality of AI-generated health information to increase accessibility and support informed decision-making among patients.

## ETHICS APPROVAL

This study was approved by Düzce University Faculty of Medicine Ethics Committee (Decision no: 2025/89, Approval Date: 24.03.2025).

## Data Availability

The datasets generated and/or analyzed during the current study are available from the corresponding author upon reasonable request.
